# EFFECTIVENESS OF A NOVEL MEDITERRANEAN DIET APP IN ENHANCING ADHERENCE: RCTS BASED ON SCT

**DOI:** 10.1093/geroni/igae098.3957

**Published:** 2024-12-31

**Authors:** Yan Su, Oleg Zaslavsky

**Affiliations:** University of Massachusetts Darmouth, Dartmouth, Massachusetts, United States; University of Washington, Seattle, Washington, United States

## Abstract

Adherence to the Mediterranean diet is a proven nutritional intervention that supports healthy aging and reduces chronic disease risk among community-dwelling older adults. Two pilot RCTs were conducted to evaluate the feasibility and efficacy of a novel mobile health intervention aimed at enhancing adherence to this diet among older users. A total of 34 participants, aged 56-88, were randomized into intervention and control groups. The intervention group used a mobile app for 3 months, receiving feedback on food choices, personalized recipe recommendations, and in-app messaging. Outcomes measured included adherence to the Mediterranean diet, five Social Cognitive Theory (SCT) constructs, and Mediterranean diet knowledge. Linear mixed models (LMMs) showed the intervention group had a significantly greater improvement of 1.52 points in Mediterranean diet adherence compared to the control group after the intervention (P<.05). The intervention group also increased their whole grain intake by 1.53-ounce equivalents more than the control group (P<.01) and their legume intake by 0.14-ounce equivalents more than the control group (P<.01). Additionally, the intervention group reduced their red meat consumption by 0.67 ounces more than the control group (P<.05). However, when each SCT construct or Mediterranean diet knowledge was included in the models individually, none were significant predictors, nor did they affect the group-time interaction, suggesting these factors did not mediate the intervention’s effectiveness. The novel mobile intervention effectively improved dietary adherence among community-dwelling older adults, likely due to the combined influence of multiple behavior change techniques or other factors not captured by SCT.

